# Supplementary material for: Differential but Concerted Expression of HSD17B2, HSD17B3, SHBG and SRD5A1 Testosterone Tetrad Modulate Therapy Response and Susceptibility to Disease Relapse in Patients with Prostate Cancer
Source: Cancers (Basel). 2021 Jul 12;13(14):3478. doi: 10.3390/cancers13143478 (PMC8303483; doi:10.3390/cancers13143478)
Supplement: Supplementary file 1 [file cancers-13-03478-s001.zip › cancers-126008-supplemental materials/cancers-1260008-Figure S1 Western Blot Raw Gel Image_Figure 5F.pdf]

cancers
MDPI
Supplemental Materials

Differential but Concerted Expression of HSD17B2, HSD17B3, SHBG and SRD5A1 Testosterone Tetrad Modulate Therapy Response and Susceptibility to Disease Relapse in Patients with Prostate Cancer

Oluwaseun Adebayo Bamodu, Kai-Yi Tzou, Chia-Da Lin, Su-Wei Hu, Yuan-Hung Wang, Wen-Ling Wu, Kuan-Chou Chen and Chia-Chang Wu

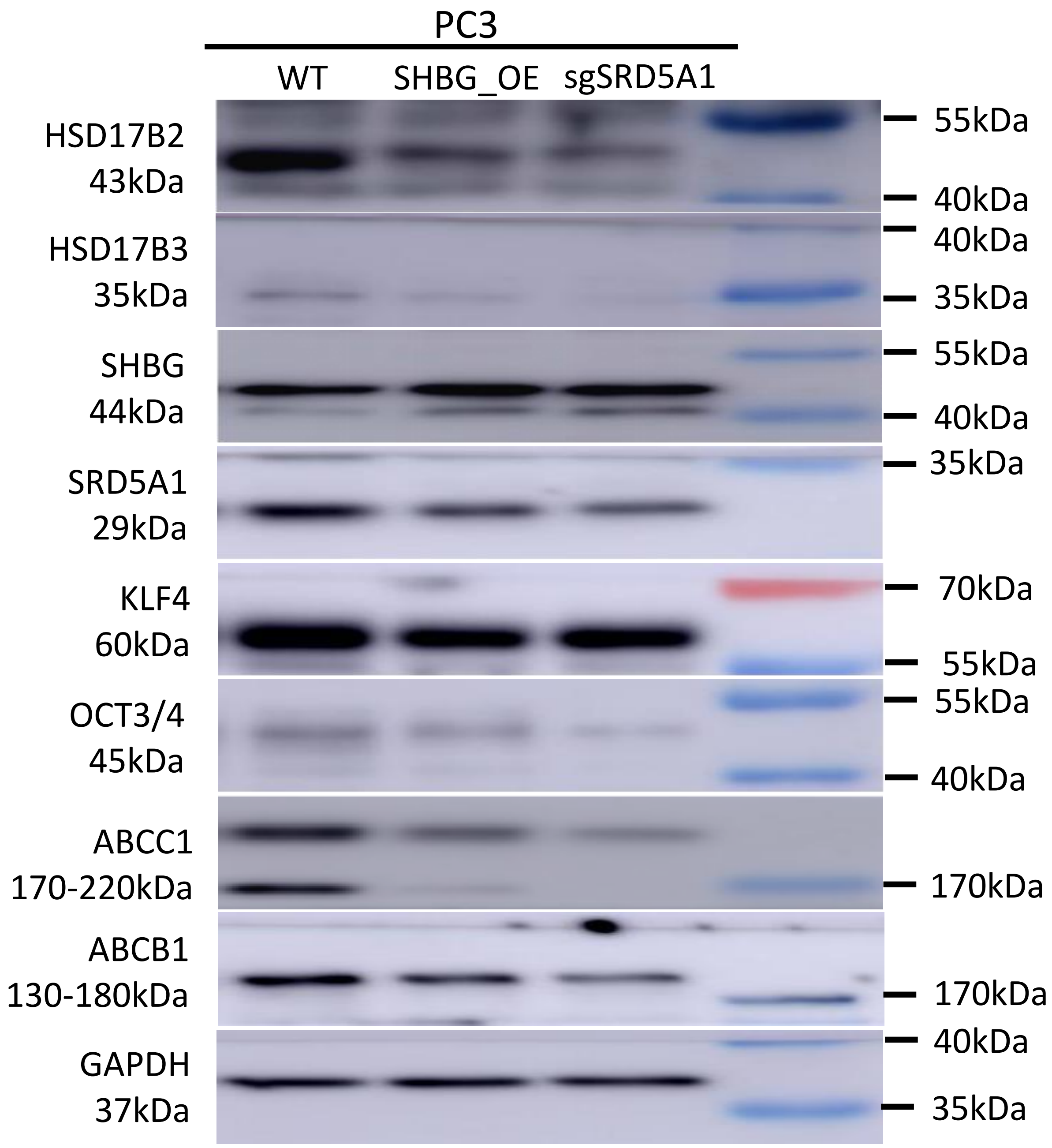

Figure S1: Original western blots of Figure 5F.
