# Supplementary material for: Differential but Concerted Expression of HSD17B2, HSD17B3, SHBG and SRD5A1 Testosterone Tetrad Modulate Therapy Response and Susceptibility to Disease Relapse in Patients with Prostate Cancer
Source: Cancers (Basel). 2021 Jul 12;13(14):3478. doi: 10.3390/cancers13143478 (PMC8303483; doi:10.3390/cancers13143478)
Supplement: Supplementary file 1 [file cancers-13-03478-s001.zip › cancers-126008-supplemental materials/cancers-1260008-supplement tables.pdf]

cancers

Supplemental Materials

Differential but Concerted Expression of HSD17B2, HSD17B3, SHBG and SRD5A1 Testosterone Tetrad Modulate Therapy Response and Susceptibility to Disease Relapse in Patients with Prostate Cancer

Oluwaseun Adebayo Bamodu, Kai-Yi Tzou, Chia-Da Lin, Su-Wei Hu, Yuan-Hung Wang, Wen-Ling Wu, Kuan-Chou Chen and Chia-Chang Wu

Table S1. Predictors effect size statistics.

	t-statistic	df	p-value	Effect size (Cohen's d)	95% Confidence Interval	
					Lower	Upper
HSD17B2	90.83	550	< 0.001	3.87	3.62	4.11
HSD17B3	163.80	550	< 0.001	6.98	6.55	7.40
SHBG	280.78	550	< 0.001	11.96	11.24	12.67
SRD5A1	646.45	550	< 0.001	27.54	25.90	29.16
Age at diagnosis	224.97	611	< 0.001	9.09	8.57	9.61
Days to first BCR	11.43	74	< 0.001	1.32	1.01	1.63
Days to new tumor event after initial treatment	12.43	63	< 0.001	1.55	1.19	1.92
OS_time	34.78	622	< 0.001	1.39	1.28	1.50
PSA value	2.64	552	0.008	0.11	0.03	0.20
BCR	8.74	533		0.38	0.29	0.47
Primary therapy outcome success	65.08	501	< 0.001	2.91	2.70	3.10
Gleason score	191.05	622	< 0.001	7.65	7.22	8.08
OS_time_5yrs	12.12	622	< 0.001	0.49	0.40	0.57

BCR, biochemical recurrence; OS, overall survival; PSA, prostate specific antigen; dF, degrees of freedom.

Table S2. Model Comparison - Predict_Recurrence_Generalized Linear Model.

Models	P(M)	P(M|data)	BF_M	BF_{10}	R^2
ENSG00000086696.9 + ENSG00000130948.8 + ENSG00000129214.13 + ENSG00000145545.10	0.010	0.515	110.524	1.000	1.000
ENSG00000086696.9 + ENSG00000130948.8 + ENSG00000129214.13 + ENSG00000145545.10 + psa_value	0.024	0.331	20.255	0.257	1.000
ENSG00000086696.9 + ENSG00000130948.8 + ENSG00000129214.13 + ENSG00000145545.10 + psa_value + age_at_diagnosis.diagnoses_yrs	0.143	0.094	0.622	0.012	1.000
ENSG00000086696.9 + ENSG00000130948.8 + ENSG00000129214.13 + ENSG00000145545.10 + age_at_diagnosis.diagnoses_yrs	0.024	0.060	2.628	0.047	1.000
Null model	0.143	0.000	0.000	0.000	0.000
ENSG00000145545.10 + psa_value + age_at_diagnosis.diagnoses_yrs	0.007	0.000	0.000	0.000	0.072
ENSG00000086696.9 + ENSG00000129214.13 + ENSG00000145545.10 + age_at_diagnosis.diagnoses_yrs	0.010	0.000	0.000	0.000	0.412
ENSG00000130948.8 + ENSG00000129214.13 + ENSG00000145545.10 + psa_value	0.010	0.000	0.000	0.000	0.913
psa_value + age_at_diagnosis.diagnoses_yrs	0.010	0.000	0.000	0.000	0.021
ENSG00000129214.13 + ENSG00000145545.10	0.010	0.000	0.000	0.000	0.318

Posterior Summaries of Coefficients - Predict_Recurrence_Generalized Linear Model.

								95% Credible Interval	
Coefficient	P(incl)	P(excl)	P(incl|data)	P(excl|data)	$BF_{inclusion}$	Mean	SD	Lower	Upper
Intercept	1.000	0.000	1.000	0.000	1.000	−6.333	1.261e −4	−6.333	−6.333
ENSG00000086696.9	0.500	0.500	1.000	0.000	∞	0.058	5.139e −5	0.058	0.058
ENSG00000130948.8	0.500	0.500	1.000	0.000	∞	0.222	7.494e −5	0.222	0.222
ENSG00000129214.13	0.500	0.500	1.000	0.000	∞	−0.358	1.271e −4	−0.358	−0.358
ENSG00000145545.10	0.500	0.500	1.000	0.000	∞	−0.213	2.293e −4	−0.214	−0.213
psa_value	0.500	0.500	0.425	0.575	0.738	−6.586e −6	9.411e −6	−2.613e −5	0.000
age_at_diagnosis.diagnoses_yrs	0.500	0.500	0.154	0.846	0.182	−7.503e −7	7.663e −6	−1.961e −5	1.399e −5

P(M), prior probabilities of the model; P(M∣data), Posterior probability of the model based on the data; BFM, Bayes factor (likelihood odds) when compared against all other models; BF10, Bayes factor (likelihood odds) when compared against the null model; R2, coefficient of determination; P(Incl), prior probabilities of inclusion of the effect in all the models; P(excl), prior probabilities of exclusion of the effect in all the models; P(Incl∣data), sum of the posterior probabilities of all models that include the effect; BFinclusion, prior to posterior odds of inclusion of a particular effect in all the considered models. Values below 1.0 are against the effect on the dependent variable. Values above 1.0 are for the effect on the dependent variable; SD, standard deviation.
